# An approach for transgender population information extraction and summarization from clinical trial text

**DOI:** 10.1186/s12911-019-0768-1

**Published:** 2019-04-09

**Authors:** Boyu Chen, Hao Jin, Zhiwen Yang, Yingying Qu, Heng Weng, Tianyong Hao

**Affiliations:** 1 School of Information Science and Technology, Guangdong University of Foreign Studies, Guangzhou, China; 2 School of Business, Guangdong University of Foreign Studies, Guangzhou, China; 3 The Second Affiliated Hospital, Guangzhou University of Chinese Medicine, Guangzhou, China; 4 School of Computer Science, South China Normal University, Guangzhou, China

**Keywords:** Gender, Transgender, Clinical trial, Information extraction, Summarization

## Abstract

**Background:**

Gender information frequently exists in the eligibility criteria of clinical trial text as essential information for participant population recruitment. Particularly, current eligibility criteria text contains the incompleteness and ambiguity issues in expressing transgender population, leading to difficulties or even failure of transgender population recruitment in clinical trial studies.

**Methods:**

A new gender model is proposed for providing comprehensive transgender requirement specification. In addition, an automated approach is developed to extract and summarize gender requirements from unstructured text in accordance with the gender model. This approach consists of: 1) the feature extraction module, and 2) the feature summarization module. The first module identifies and extracts gender features using heuristic rules and automatically-generated patterns. The second module summarizes gender requirements by relation inference.

**Results:**

Based on 100,134 clinical trials from ClinicalTrials.gov, our approach was compared with 20 commonly applied machine learning methods. It achieved a macro-averaged precision of 0.885, a macro-averaged recall of 0.871 and a macro-averaged F_1_-measure of 0.878. The results illustrated that our approach outperformed all baseline methods in terms of both commonly used metrics and macro-averaged metrics.

**Conclusions:**

This study presented a new gender model aiming for specifying the transgender requirement more precisely. We also proposed an approach for gender information extraction and summarization from unstructured clinical text to enhance transgender-related clinical trial population recruitment. The experiment results demonstrated that the approach was effective in transgender criteria extraction and summarization.

## Background

Clinical trials are observations or experiments carried out in clinical research. Under efficacious guidance and strict administration, they can generate reliable testimony and contribute remarkably to evidence-based medicine [1, 2]. To a large extent, obtaining satisfactory outcomes from clinical trials depends on the effectiveness of identifying and recruiting suitable participants [3, 4]. However, the recruitment of target population has turned into a main impediment in clinical trials due to the time-consuming labor, great cost of fund, and rising complexity [5–9]. The unsatisfactory result of recruitment can bring tremendous trouble to clinical investigators and omitted opportunities to patients [4, 10–12].

In the recruiting phase, eligibility criteria evaluate the qualification of participants for certain clinical care or research. Gender is a widely-used and core criterion for electronic prescreening and the gender requirement is approximately defined as structured information by every clinical trial [13–15]. Besides, gender is widely utilized for clinical data searching and processing, e.g., the gender identification in Electronic Medical Record (EMR) data [16], the transgender status estimation using EMR data [17], and the core data element for clinical retrieval [18]. Thus, the explicit gender type requirement is essential for enrolling proper participants in clinical trials.

However, prevalent applied gender-required criteria in clinical trial may contain gender issues including incompleteness and ambiguity issues, particularly for transgender-recruiting trials. Transgender is an umbrella term denoting the population whose gender identity or expression is different from their assigned sex at birth [19, 20]. Existing websites for registry of clinical trials only define *Male*, *Female* and *Both* as structured information in the gender requirement. Whereas, the transgender type is generally neglected in gender specification during registration.

Taking ClincialTrials.gov,^1^ the largest official registry of clinical trials in the world, as an example. ClinicalTrials.gov, sponsored by the United States National Institutes of Health (NIH), provides a summary for each registered clinical trial [21]. In this registry, clinical trials recruiting certain types of transgender population need to clarify gender requirements in sex eligibility section. Nevertheless, only “*Male*”, “*Female*”, and “*All*” are listed as the structured gender types alternatives for trial registration, which may induce unsuitable recruitment for transgender population. For example, in the clinical trial NCT01880489,^2^ eligible participants need to be transgender female according to “*Identify as a transgender woman (assigned male at birth and currently identify as female)*” from inclusion criteria section. However, the gender requirement is incorrectly registered as “*Female*” in the structured section “*Sexes Eligible for Study*” due to lack of the “*transgender*” option on the website. For trials recruiting transgender populations (e.g., clinical trials studying Human Immunodeficiency Virus), the incorrect transgender information issue is much common. As a result, this incorrect gender registration may cause wrong information in clinical trial text processing and negative influences in clinical trial electrical recruitment.

**Fig. 1:**
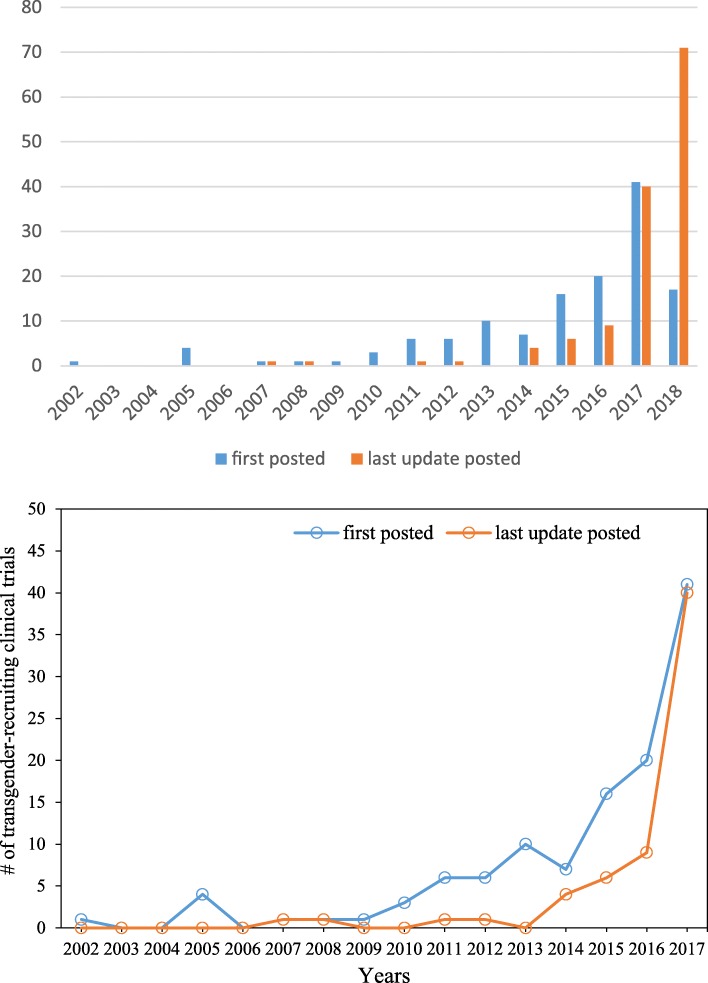
The number of clinical trials recruiting transgender population on ClincialTrial.gov

Meanwhile, the population of transgender and the number of trials requiring for transgender has been increasing. As reported in 2011, around 700,000 transgender adults (about 0.3% of total adult population) were identified in the United States [22]. This number rose rapidly to almost 1.4 million in 2016 [23] and was nearly twice as high as the number in 5 years ago. To explore the demographic changes of transgender population in clinical trials, we conducted a manual investigation on ClinicalTrials.gov. We used keyword searching combined with manual review to identify transgender-recruiting trials from all 277,012 clinical trials on ClinicalTrials.gov as to 2018/07/10. With respect to the “*first posted*” and “l*ast update posted*” dates of the clinical trials during 2000 to 2017, the number of trials recruiting transgender population was calculated and reported in Fig. 1, demonstrating an upward trend. This growth trend illustrates the increasing importance of the identification of transgender-recruiting trials for transgender population in the participation of clinical trials. Therefore, the data quality issue caused by inappropriate gender registration may lead to more incorrect transgender population recruitment cases among clinical trial studies. Thus, it is imperative and urgent to deal with the transgender data quality issues.

To that end, focusing on improving transgender related-trials searching and recruiting, we designed a virtual gender model to present more complete and explicit gender requirement information. In addition, an automated approach was developed for transgender information extraction and summarization from unstructured clinical trial text. This approach consists of the feature extraction module and the feature summarization module. The feature extraction module utilizes patterns automatically learned from annotated clinical trial text and combines with a list of heuristic rules to extract gender features from clinical trials. The second module computes these features and summarizes gender requirements by relation reasoning for a clinical trial.

We further treated the whole procedure of gender information extraction and summarization as a multi-classification task and compared our approach with 20 machine learning methods on the same clinical text datasets, which incorporate transgender-recruiting trials and non-transgender-recruiting trials together. Based on the largest dataset containing 100,134 trials, our approach achieved a macro-averaged precision of 0.885, a macro-averaged recall of 0.871 and a macro-averaged F_1_-measure of 0.878. The results outperformed all the other baseline methods and demonstrated the effectiveness of the approach in gender information extraction and summarization.

## Methods

**Fig. 2:**
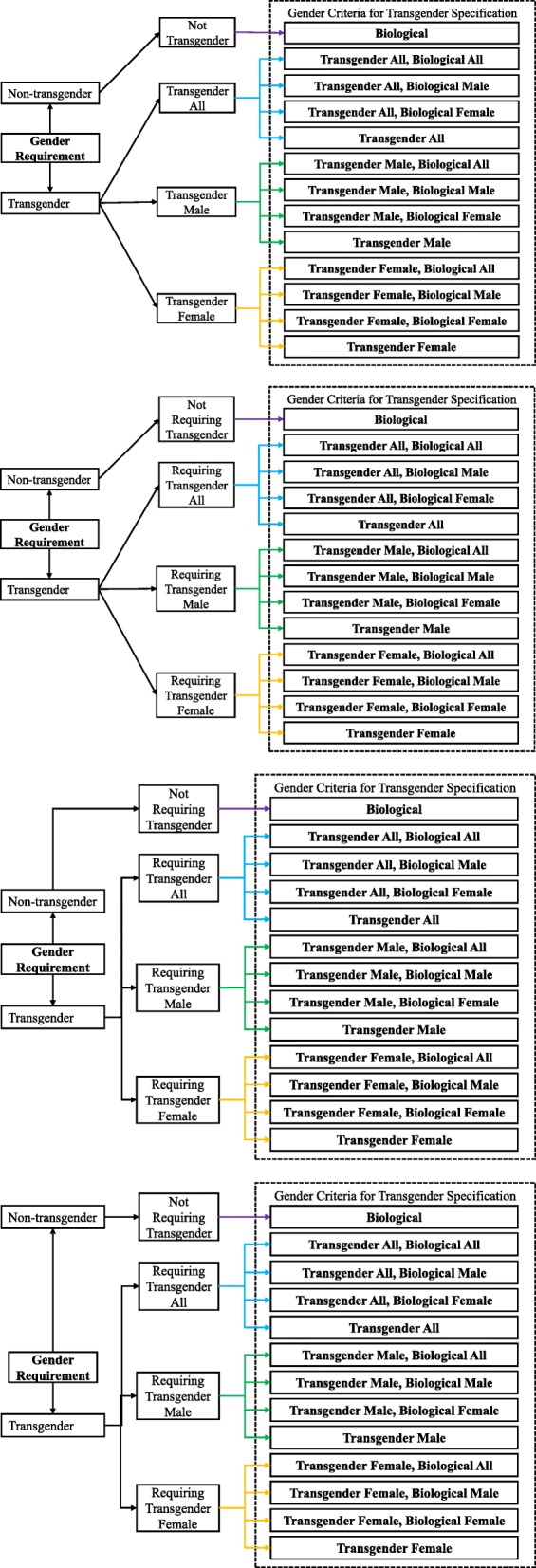
The virtual gender model and its mapping relations with conventional gender types

To solve the transgender-related issues, a virtual gender model is proposed, as shown in Fig. 2. This model extends the widely and commonly used conventional gender types in ClinicalTrials.gov registration options (‘*Male*’, ‘*Female*’, and ‘*All*’). To include transgender types, 13 specific gender criteria types are defined. Since the model mainly focuses on transgender criteria problem, all the non-transgender-recruiting types are assigned as one type [‘*Biological*’]. The type [‘*Transgender All, Biological All*’] represents that the gender criteria type in the clinical trial aims to recruit the populations with transgender male & female and biological male & female. Similarly, the reminder 11 types are [‘*Transgender All, Biological Male*’], [‘*Transgender All, Biological Female*’], [‘*Transgender All*’], [‘*Transgender Male, Biological All*’], etc.

**Fig. 3:**
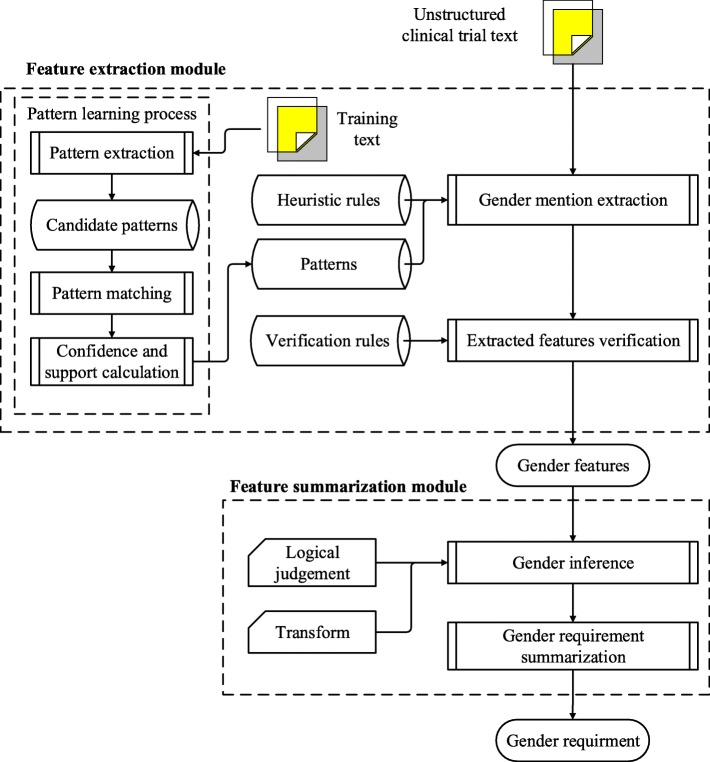
The framework of our approach

Based on the gender model, we further propose an automated approach for extracting and summarizing gender information from free clinical trial text. This approach comprises the feature extraction module for extracting gender features and the feature summarization module for concluding final gender requirements based on the proposed gender model. The overall framework of the approach is shown as Fig. 3.

### Feature extraction

Clinical trial text frequently contains gender mentions. The feature extraction module utilizes a group of heuristic rules to identify, extract and verify gender information features from clinical trial text including “Study Description” and “Eligibility Criteria” sections. These rules consist of predefined logical relations, a list of gender mention features from clinical trials, and regular expressions. Then, a set of structural patterns is automatically generated from clinical text based on transgender mention annotations. After that, the heuristic rules and the patterns are combined to be applied to gender information extraction and verification from clinical trial text.

#### Heuristic rule generation

According to the investigation of existing clinical trial text, a variety of gender mention features is categorized into distinct gender mention types. We treat the gender mention detection as a process of feature identification. The examples of some gender mention types and the corresponding features are listed as Table 1. For instance, the feature extraction module regards features ‘*males*’, ‘*male*’, ‘*man*’, ‘*men*’, ‘*gay*’, ‘*gays*’ and ‘*masculine*’ as the gender mention type ***[Male]***.

**Table 1:** The gender mention types and their related gender mention features

Gender mention types	Gender mention features
*[Male]*	*‘males’, ‘male’, ‘man’, ‘men’, ‘gay’, ‘gays’, ‘masculine’*
*[Female]*	*‘females’, ‘female’, ‘woman’, ‘women’, ‘lesbians’, ‘lesbian’, ‘les’, ‘feminine’*
*[Two_Gender]*	*‘m/f’, ‘m&f’, ‘both genders’, ‘two genders’, ‘two-gender’, ‘all genders’, ‘all-gender’*
*[Biological]*	*‘biologically’, ‘biological’, ‘cisgender’*
*[Transgender]*	*‘transgender’, ‘transsexual’, ‘transsexuals’, ‘transsexualism’, ‘change sex’, ‘changed sex’, ‘sex changed’, ‘change gender’, ‘changed gender’, ‘gender changed’, ‘transgendered’*
*[Male_Abbreviation]*	*‘msm’, ‘msw’, ‘msm/w’, ‘msw/m’, ‘ymsm’*
*[Partner]*	*‘partner’, ‘partners’, ‘sexual partner’, ‘sexual partners’, ‘wife’, ‘husband’*
*[Negation_Word]*	*‘no’, ‘not’, ‘except’, ‘besides’, ‘rather’, ‘rather than’, ‘neither’, ‘not identify as’, ‘not identified as’*

Combining with the defined gender mention types, we develop a set of heuristic rules to detect the mentions in clinical trial text. The rules contain logical relations such as “*If (gender X) in exclusion criteria, then output (Not gender X)*” and regular expressions such as “*match ‘*(*[Transgender] [Female]*) *or* (*[Female] [Transgender]*)*’ as <Transgender Female>*”, where ‘*[Transgender]*’ and ‘*[Female]*’ are two pre-defined gender mention types. Given a sentence “*Self identify as a transgender woman*” (NCT03270969^3^), the gender mention “*transgender woman*” is identified using the regular expression and is annotated as “*<Detected Gender Mention = Transgender Female>*”.

#### Transgender pattern learning

Identifying transgender features by utilizing heuristic rules only may lead to erroneous extraction. For instance, in sentence “ *… Participants who were female at birth, who now identify as male, will not be excluded …* ” (NCT02356302^4^), the “*male*” indicates transgender male rather than biological male from the context. However, it is wrongly regarded as not transgender features by heuristic rule as this sentence does not contain any transgender-specific key words. Thus, we incorporate automatic structural pattern learning to improve feature extraction performance. The essential notion of the pattern learning is to extract all pattern candidates based on original annotated text and to filter patterns which may have less significance to feature extraction.

To automatically generate the transgender matching patterns, the clinical trial text with transgender feature annotations is split into sentences initially. The feature extraction module leverages a sentence boundary identification algorithm by rule-based matching to split text into individual sentences, especially for the text lacking full stop symbols. Those sentences are verified by pre-defined rules to rectify incorrect cases, such as treating the “*.*” in “e.g.*,*” as stop symbols. Then, the original transgender mention annotation tags are replaced with a specified tag “*<TG_Start > <TG_End>*” for context extraction. For example, a sentence is processed as “ *… Participants who were female at birth, <TG_Start>who now identify as male<TG_End>, will not be excluded …* ” (NCT02356302^5^).

After replacement, a list of pattern candidates is extracted by setting a word window length as a parameter *β*. The optimized value of *β* is empirically chosen as 7, presenting that the number of words around the transgender tag is not larger than 7. All patterns containing the tags and their surrounding words are extracted and regarded as candidate patterns. Each candidate pattern is further matched back to the training clinical trial text. For example, the pattern “*female at birth, <TG>, will not*” can be matched with “ *… Participants who were female at birth, <TG_Start>who now identify as male<TG_End>, will not be excluded …* ” (NCT02356302^6^), “ *… Participants who are female at birth, <TG_Start>who now identify as male<TG_End>, will not be excluded …* ” (NCT03467347^7^^7^) and “*… Participants who are female at birth, <TG_Start > who now identify as male < TG_End>, will not be excluded …*” (NCT03234400^8^). The confidence and support values of each candidate pattern are calculated after matching. We define a support metric *∂*_*S*_ as the count of correct matches and a confidence metric *∂*_*C*_ as the rate of correct expression matches among all matches. The candidate patterns with confidence or support values lower than the two metrics are regarded as invalid patterns and are filtered out. To enlarge the matching coverage of the generated patterns, *∂*_*C*_ is set as 0.7 and *∂*_*S*_ is set to 4 empirically in this study.

#### Gender mention verification

The automatically learned patterns and heuristic rules are utilized to identify gender mention features from free clinical trial text sequentially. After the detection, the mentions are identified and annotated with corresponding gender types. A context-based method is then proposed to verify the gender mention annotations. In certain cases, some identified mentions are not adaptive to context information and should be excluded. For example, the “*male*” identified in “*male sex partners*” (NCT02704208^9^) and the “*female*” identified in “*Male Patients with female sexual partners*” (NCT00231465^10^) do not represent required target population and should be removed from the annotations. Therefore, we develop a list of regular expressions to verify the annotations. The rule “*<Detected Gender Mention>* (***[Partner]***)” thus is used to detect the identified “*male*” and “*female*” in similar examples.

In addition, some identified mentions are associated with negation words, which may change the meaning of required gender types. To detect and rectify those negation cases, a list of negation features as *[Negation_Word]* is defined, as listed in Table 1. The module identifies the negation features and filters out irrelevant gender mentions in sentence-level context. For example, the rule “*[Negation_Word] <Detected Gender Mention>*” can be used to identify “*transgendered*” in “*Biologically male (not transgendered)*” (NCT01023620^11^), where “transgendered” should be annotated together with “*not*”.

Algorithm 1 illustrates the feature extraction module for extracting gender information. Firstly, an unstructured clinical trial text is split into sentences. The algorithm then detects and extracts gender mentions from each sentence by incorporating the generated transgender patterns and heuristic rules. At this step, we apply the patterns from *Generated_Patterns* and heuristic rules from *Heuristic_Rules* to annotate gender features from each sentence. *Generated_Patterns* are patterns generated automatically from clinical trial text with manual annotations, as shown in line 5. Then rules from *Verification_Rules* are used to remove marked mentions that should not be included. Those extraction and verification procedures are shown as line 7–22.

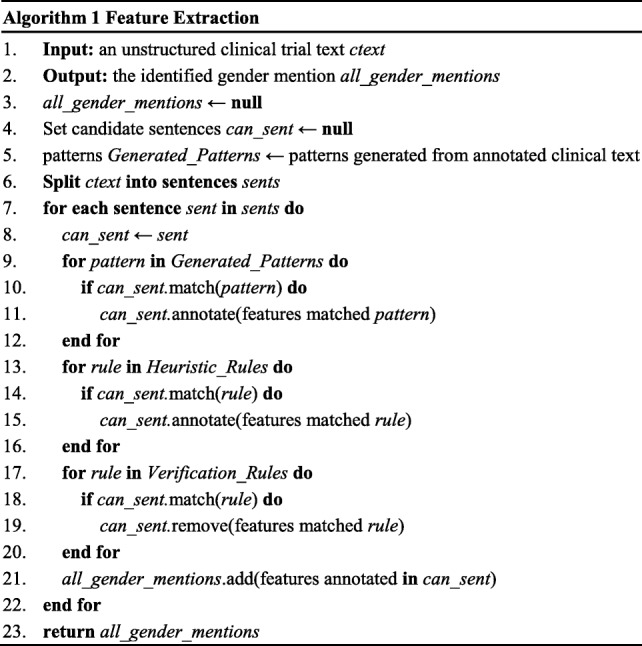



### Gender summarization

#### Gender mention inference

Since different gender types have internal relations, we design a list of gender inference functions for relation calculation and deduction. These functions are composed of logical judgment functions and transformation functions.

The logical judgment function determines whether gender types are conformed to a certain relation. Some examples of logical judgment functions are shown as Table 2 and each function contains a function description in logical way. For example, we used *SuperJudgement* function to determine whether a gender type has superior relation with the other or another. Using this function, ‘*Transgender All*’ can be treated as superior gender of ‘*Transgender Male*’ or ‘*Transgender Female*’, and the model can therefore compute the relations among those gender types.

**Table 2:** Examples of logical judgment functions and their descriptions

Function name	Description	Example
*SubJudgement* (*G*_*1*_*, G*_*2*_)	**If** *G*_*1*_ is subordinate gender of *G*_*2*_**:** return **True** **Else:** return **False**	*G1 = ‘Transgender Male’* *G2 = ‘Transgender All’* Return **True**
*SuperJudgement* (*G*_*1*_*, G*_*2*_)	**If** *G*_*1*_ is superior gender of *G*_*2*_**:** return **True** **Else:** return **False**	*G1 = ‘Transgender All’* *G2 = ‘Transgender Male’* Return **True**
*ReverseJudgement* (*G*_*1*_*, G*_*2*_)	**If** *G*_*1*_ is **NOT** *G*_*2*_**:** return **True** **Else:** return **False**	*G1 = ‘Transgender Female’* *G2 = ‘Transgender Female’* Return **False**
*SimilarJudgement* (*G*_*1*_*, G*_*2, …*_)	**If** *G*_*1*_, *G*_*2*_, _*…*_ is similar types**:** return **True** **Else:** return **False**	*G1 = ‘Transgender All’* *G2 = ‘Transgender Male’* Return **True** *G1 = ‘Transgender All’* *G2 = ‘Biological Male’* Return **False**
*SplitJudgement* (*G*_*1*_)	**If** *G*_*1*_ can be split**:** return **True** **Else:** return **False**	*G1 = ‘Transgender All’* Return **True** *G1 = ‘Transgender Male’* Return **False**

With the logical judgment functions, we further develop a list of transformation functions to operate and transform different gender types for concluding final gender requirements. The examples of transformation functions are shown as Table 3, where some of them contain parameter restrictions of logical judgment functions. For example, ‘*Transgender All*’ can be split into ‘*Transgender Male*’ and ‘*Transgender Female*’ using the function *Split*(‘*Transgender All*’). ‘*Biological Male*’ and ‘*Biological Female*’ can be merged into ‘*Biological All*’ using the function *Merge*(‘*Biological Male*’, ‘*Biological Female*’). ‘*Biological Female*’ can be converted into ‘*Transgender Male*’ using the function *TransConstrain*(‘*Biological Female*’). The function *TransConstrain* can be applied to transform *biological gender* into transgender types while the context is identified as transgender condition.

**Table 3:** Examples of transformation functions and their descriptions

Function	Description	Parameter Restriction	Example
*Split*(*G*_*1*_)*→* (*G*_*2*_*, G*_*3*_)	*Splitting G*_*1*_ *into G*_*2*_ *and G*_*3*_	*SplitJudgement*(*G*_*1*_) *==* **True**	**Input** *G*_*1*_ *=* ‘*Transgender All*’ **Ouput** *G*_*2*_ *=* ‘*Transgender Male*’ *G*_*3*_ *=* ‘*Transgender Female*
*Merge*(*G*_*1*_*, G*_*2*_) *→G*_*3*_	*Merging G*_*1*_ *and G*_*2*_ *into G*_*3*_	*SplitJudgement*(*G*_*1*_) *==* **False** *SplitJudgement*(*G*_*2*_) *==* **False** *SimilarJudgement*(*G*_*1*_*, G*_*2*_) *==* **True** *ReverseJudgement*(*G*_*1*_*, G*_*2*_) *==* **True**	**Input** *G*_*1*_ *=* ‘*Biological Male*’ *G*_*2*_ *=* ‘*Biological Female*’ **Ouput** *G*_*3*_ *=* ‘*Biological All*’
*TransConstrain* (*G*_*1*_) *→ G*_*2*_	*G*_*1*_ *is transformed into the transgender type G*_*2*_		**Input** *G*_*1*_ *=* ‘*Biological Male*’ **Ouput** *G*_*2*_ *=* ‘*Transgender Female*’

#### Gender requirements summarization

To conclude the required gender types of a clinical trial, all the mapped gender types with valid annotations are split into a list of meta gender types, i.e., ‘*Biological Male*’, ‘*Biological Female*’, ‘*Transgender Male*’ and ‘*Transgender Female*’ according to the gender relations defined in the feature summarization model. For example, ‘*Biological All*’ is split into ‘*Biological Male*’ and ‘*Biological Female*’.

Since a text may contain multiple gender types while some of them may be noise, we design a strategy using majority rule to detect frequently mentioned gender types considering that some meta gender types are predominant in a text. All meta gender types are then sorted by their frequencies in descending order. If the frequency of a meta gender type ranked at top *i* + 1 multiplies a threshold *μ* is lower than the previous meta gender type ranked at *i*, the feature summarization module treats the meta gender types from *i* + 1 to *n* as noise. Using *MG*_*i*_ to present the frequency of a meta gender type ranked at top *i* and *μ* to denote the threshold, the final predominant score as *Pred* is calculated using Eq. (1). If *Pred* < 1, the module treats (*MG*_*1*_, … *MG*_*i*_) as predominant gender types and treats the reminder as noise. The optimization of *μ* is presented in Experiment and Result section.

1 $$ Pred=\frac{MG_{i+1}}{MG_i}\times \mu $$

Taking clinical trial NCT02401867^12^ as an example. The study description contains the statement “*among sexually active female-to-male (FTM) transgender adults*”, “*among 150 FTM patients in Boston*”, “*online focus groups with FTMs*” and “*to gather information on the sexual health needs of FTM individuals*”. The study population description contains the statement “*enroll 150 female-to-male (FTM) individuals*”, “*recruited from the existing FTM patient population*” and “*recruit 40% racial/ethnic minority FTMs*”. The inclusion criteria includes “*Assigned a female sex at birth and now self-identifies as a man, trans masculine, trans man, FTM, transgender, genderqueer/non-binary, transsexual, male, and/or another diverse transgender identity or expression*”. After gender information extraction and verification of the above text, the identified and valid ‘*Transgender Male*’ type occurs 10 times, while ‘*Transgender All*’ twice, ‘*Biological Male*’ twice and ‘*Biological Female*’ once. After splitting ‘*Transgender All*’ into two meta gender types, ‘*Transgender Male*’ is counted as 12 times and ‘*Transgender Female*’ as 2. According to the Eq. (1), ‘*Transgender Male*’ is treated as *MGT*_*1*_ while ‘*Transgender Female*’, ‘*Biological Male*’ and ‘*Biological Female*’ are treated as *MGT*_*2*_, *MGT*_*3*_ and *MGT*_*4*_. By using the threshold *μ* as 5, the *Pred* of *MGT*_*1*_ is lower than 1. Therefore, the feature summarization module takes ‘*Transgender Male*’ as the predominate gender type and ignores the rest gender types.

The module treats all kept meta gender types as equal and merges them using the transformation function *Merge*(*G*_*1*_*, G*_*2*_) for generating a final gender conclusion. For example, the meta gender types ‘*Transgender Female*’ and ‘*Transgender Male*’ are merged into the finial gender ‘*Transgender All*’.

Algorithm 2 defines the feature summarization module for concluding transgender-requiring clinical trials. All the extracted gender-related mentions *all_gender_mentions* from Algorithm 1 are transformed into meta genders *MetaGenders* by the gender inference functions. The *MetaGenders* are then sorted by their count of occurrences in text in descending order, as presented in Line 6. The final gender requirement summary is obtained based on the result of comparison between *MetaGender* [*i*] and *MetaGender* [*i* + 1]*threshold, where the steps are shown as line 7–11.

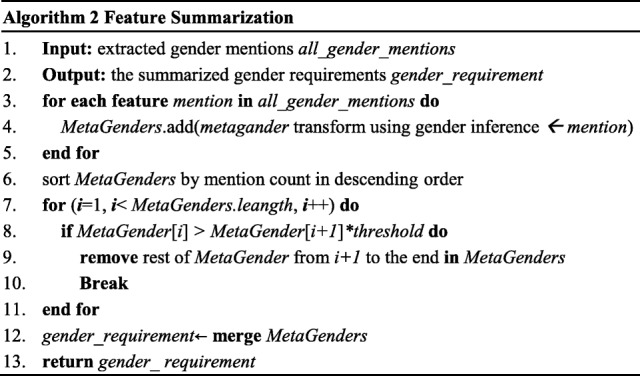



## Experiment and result

### Evaluation metrics

For performance evaluation, we treat the gender information identification and summarization as a multi-classification task. As commonly used as performance evaluation metrics in Nature Language Processing (NLP) and information retrieval tasks, precision, recall and F_β_-measure are adopted in the experiment [24, 25]. Typically, in a binary classification task, a data is labeled as either positive or negative (where positive and negative represent two generic categories). A confusion matrix can be generated according to True Positive (*TP*), True Negative (*TN*), False Positive (*FP*), and False Negative (*FN*). In the matrix, precision represents the percentage of correctly classified positive data divided by the total number of data classified as positive (*Precision* = *TP*/(*TP* + *FP*)). Recall is the percentage of correctly classified positive data divided by the total number of data expecting to be classified as positive (*Recall* = *TP*/(*TP* + *FN*)). F_β_-measure is the harmonic mean of precision and recall (Eq. 2). Non-negative real value *β* enables F_β_-measure to balance emphasize precision or recall. We empirically use F_1_-measure by setting *β* = 1 to equal the weights of precision and recall.

2 $$ {F}_{\beta }- measure=\frac{\left(1+{\beta}^2\right)\times Precision\times Recall}{\left(\ {\beta}^2\times Precision\ \right)+ Recall} $$

In addition, the proportion of non-transgender-recruiting clinical trials is much higher than transgender-recruiting trials. The results of precision, recall, and F_1_-measure may be affected by such an unbalanced data. We thus use micro-averaged values as additional metrics to reduce the effect of unbalanced quantity of predominated gender types. The macro-averaged metrics assign equal weights to categories in the evaluation to discount the performance of better-populated categories [24, 25]. The calculations of macro-averaged precision, macro-averaged recall, and macro-averaged F_1_-measure are shown as Eqs. 3, 4, and 5, respectively, where *n* denotes the number of gender types.

3 $$ {Precision}_{macr\mathrm{o}}=\frac{1}{n}\sum \limits_{i=1}^n{Precision}_i $$

4 $$ {Recall}_{macro}=\frac{1}{n}\sum \limits_{i=1}^n{Recall}_i $$

5 $$ {F}_1-{measure}_{macro}=\frac{2\ast {Precision}_{macro}\ast {Recall}_{macro}}{Precision_{macro}+{Recall}_{macro}} $$

### Dataset

The 277,012 clinical trials on the ClinicalTrials.gov as to 2018/07/10 were used as experimental data. All transgender-related keywords were used to match the trial text to retrieve transgender-recruiting clinical trials as a candidate dataset. Three human annotators including one clinician and two clinical researchers manually annotated the dataset independently using the proposed gender data model. The inter-agreement rate was 73% using Fleiss Kappa. After discussion, the three annotators solved all disagreements and formed the final gold standard for transgender criteria in clinical trials. As a result, 134 clinical trials were identified as transgender-recruiting trials, generating a dataset **TG**.

To generate transgender annotation dataset for automated transgender patterns learning, we leveraged a bootstrap method which reduced the impact of dataset size difference and increase the efficiency of experimental estimation [26]. The bootstrap method is useful when the scale of dataset was not large and effectively partitioning training sets was difficult [26]. Based on the dataset **TG**, we used a bootstrap method to generate a transgender dataset **TG’**. One trial from **TG** was randomly selected and its copy was sent into **TG’**. This execution will repeat until the scale of **TG’** is equal to **TG**. Then, we randomly extracted 10,000 clinical trials containing non-transgender-related features. These clinical trials were added into **TG’** to form transgender patterns learning training dataset with better validation by enlarging data scale. Based on transgender patterns learning training dataset with manually annotated transgender features, our approach extracted all pattern candidates from this dataset and calculated the confidence and support by matching back to original annotated training dataset. After calculation, the patterns with a confidence and a support lower than a threshold were filtered out. As a result, the approach generated 14 patterns.

To expend the experiment dataset for better evaluating the performance of our approach, we randomly extracted 5000, 10,000, 20,000, 40,000, 60,000, 80,000 and 100,000 non-transgender-recruiting trials and combined into the dataset **TG** respectively to form seven datasets: dataset **A**(134 transgender-recruiting trials + 5000 non-transgender-recruiting trials), dataset B(134 transgender trials-recruiting + 10,000 non-transgender-recruiting trials), dataset C(134 transgender trials-recruiting + 20,000 non-transgender-recruiting trials), dataset D(134 transgender trials-recruiting + 40,000 non-transgender-recruiting trials), dataset E(134 transgender-recruiting trials + 60,000 non-transgender-recruiting trials), dataset F(134 transgender-recruiting trials + 80,000 non-transgender-recruiting trials), and dataset **G**(134 transgender-recruiting trials + 100,000 non-transgender-recruiting trials). *k*-fold cross-validation strategy was used in the evaluation and *k* was set as 10 empirically.

### Result

To optimize the threshold *μ* described in the Method section, the performances in terms of F_1_-measure values were calculated by setting the threshold from 1 to 10 leveraging 10-fold cross-validation. Taking dataset **G** as an example, as shown in Table 4, the results showed that the F_1_-measure obtained the different values when the threshold increased from 1 to 10 in round 1 to 10 on the training datasets (nine of ten using 10-fold), respectively. We thus selected *μ* = 5 in round 1–3 and 5–10 while *μ* = 4 in round 4 as the optimized parameters. Eventually, *μ* = 5 was chosen as the best parameter value for the following experiments.

**Table 4:** The parameter training using F_1_-measure with three-fold cross-validation

*μ*	Round 1	Round 2	Round 3	Round 4	Round 5	Round 6	Round 7	Round 8	Round 9	Round 10
1	0.162	0.163	0.174	0.157	0.159	0.162	0.162	0.158	0.157	0.156
2	0.599	0.563	0.659	0.599	0.611	0.618	0.555	0.607	0.591	0.616
3	0.741	0.724	0.830	0.747	0.726	0.769	0.731	0.745	0.748	0.763
4	0.866	0.849	0.853	**0.873**	0.859	0.888	0.863	0.877	0.872	0.879
5	**0.873**	**0.855**	**0.857**	0.869	**0.864**	**0.892**	**0.867**	**0.886**	**0.878**	**0.882**
6	0.854	0.835	0.834	0.846	0.844	0.873	0.855	0.880	0.858	0.861
7	0.838	0.820	0.834	0.828	0.830	0.858	0.840	0.860	0.843	0.843
8	0.838	0.822	0.836	0.829	0.831	0.859	0.842	0.862	0.845	0.845
9	0.838	0.822	0.836	0.829	0.831	0.859	0.842	0.862	0.845	0.845
10	0.840	0.824	0.837	0.831	0.833	0.861	0.844	0.863	0.847	0.845

**Fig. 4:**
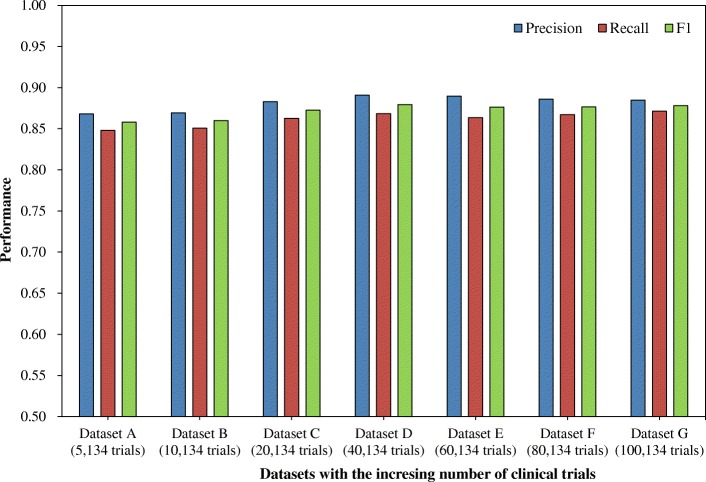
The performance of our approach on different datasets

To test the stability of our approach, it ran on all the datasets **A** to **G**. The macro-averaged precision, recall and F_1_-measure in each round were calculated. The values were further averaged based on ten rounds and were reported in Fig. 4. The macro-averaged precision values were 0.868, 0.869, 0.883, 0.891, 0.89, 0.886 and 0.885; the macro-averaged recall values were 0.848, 0.851, 0.863, 0.868, 0.863, 0.867 and 0.871; and the macro-averaged F_1_-measure values were 0.858, 0.860, 0.873, 0.879, 0.876, 0.876 and 0.878. Since the seven datasets reflected the increasing number of clinical trials (from 5134 trials to 100,134), the macro-averaged precision, recall and F_1_-measure values had 0.8, 0.8 and 0.6% variance on the dataset **C** to **G**. Based on the largest dataset **G**, the approach achieved a macro-averaged precision of 0.885, a macro-averaged recall of 0.871, and a macro-averaged F_1_-measure of 0.878.

In addition, to compare our approach with state-of-the-art methods, we applied 20 widely used machine learning algorithms as baselines. These baselines were implemented in a suite of machine learning toolkit - Weka 3.8 [27], including Bayesian Network [28], Naive Bayes [29], SMO (Sequential Minimal Optimization) [30], Random Forest [31], LMT (Logistic Model Tree) [32], and J48(C4.5) [33]. The same features were processed by those baseline algorithms in WEKA using the same 10-fold cross-validation strategy. Our approach was compared with those algorithms using macro-averaged F_1_-measure on the dataset **A** to **G. The performance** greater than 0.6 **in terms of** macro-averaged F_1_-measure were reported in Table 5. Classical Random Forest, LMT, and Bayes Net achieved the macro-averaged F_1_-measure 0.765, 0.665 and 0.655 on dataset **G**, respectively. Our approach achieved the highest macro-averaged F_1_-measure score, outperforming all the baselines on every dataset.

**Table 5:** The performance comparison on the datasets (A to G) using Macro-averaged F_1_-measure

Method	A	B	C	D	E	F	G
Logit Boost	0.637	0.674	0.681	0.639	0.667	0.636	0.628
Logistic	0.745	0.735	0.693	0.667	0.678	0.706	0.646
Bayes Net	0.680	0.662	0.652	0.624	0.665	0.665	0.655
Simple Logistic	0.761	0.668	0.697	0.684	0.644	0.685	0.658
LMT	0.772	0.668	0.643	0.686	0.625	0.686	0.665
Random Committee	0.728	0.738	0.696	0.695	0.688	0.750	0.673
Decision Table	0.637	0.609	0.590	0.599	0.605	0.617	0.675
Random Tree	0.674	0.667	0.661	0.646	0.652	0.668	0.718
Random Forest	0.774	0.739	0.760	0.698	0.733	0.747	0.765
Our approach	**0.858**	**0.860**	**0.873**	**0.879**	**0.876**	**0.876**	**0.878**

## Discussion

Our approach was proposed for automatically extracting and summarizing transgender information from unstructured clinical trial text. On the basis of our previous work at [34], we improved the transgender extraction method by introducing an automatic pattern learning method. Compared with the previous work, the new approach intentionally applied the macro-averaged metric in order to better validate the approach considering that the experiment datasets contain much less transgender-recruiting trials than non-transgender-recruiting trials. Besides, the new approach was compared with 20 commonly applied machine learning algorithms on the same experiment datasets and achieved higher performance. The overall micro-averaged F_1_-measure and macro-averaged F_1_-measure of our approach on the largest dataset **G** was 0.98 and 0.878, respectively, achieving 0.02 and 0.113 higher compared with the best baseline algorithm Random Forest. According to the results, the approach could remain stable when the number of clinical trials was increasing.

To demonstrate the effectiveness of integrating pattern-learning method, we compared the performance of our approach with or without pattern matching on the dataset **A** to **G**. The macro-averaged F_1_-measure values without pattern matching were 0.791, 0.804, 0.801, 0.807, 0.813, 0.803 and 0.813 respectively. The results illustrated that the performances of the approach with pattern matching were consistently higher than using heuristic rule only.

To understand the weakness of our approach for further improvement, we analyzed all error cases and identified the following error types:

1. **Context verification errors:** The incorrect gender mention identifications incurred when context containing irrelevant information. For example, in “*The specific objectives of this study are reduce stigma towards lesbian, gay, bisexual, and transgender persons in Swaziland and Lesotho*” (NCT02410434^13^), the “*lesbian, gay, bisexual, and transgender persons*” was annotated as [‘*Transgender All, Biological All*’] by the approach, while human annotators treated it as irrelevant information. In “*this is a process that provides an opportunity to study the sex hormone dependent influences that explain differences in morbidity in men and women respectively*” (NCT02518009^14^), the approach treated “*men and women*” as [“*Biological Both*”] while human annotators treated it as irrelevant information.
2. **Pattern matching errors:** While matching the correct features, the pattern might also identify the wrong information. For instance, the pattern “*with men (msm) and <TG> (*” correctly identified the transgender feature “*transgender women*” in “ *… thai men who have sex with men (msm) and transgender women (tg) …* ” (NCT01869595^15^). However, this pattern incorrectly matched the non-transgender information “*female sex workers*” in “ *… including early injectors, men who have sex with men (msm) and female sex workers (fsw) …* ” (NCT02573948^16^). We intend to open the source code of the proposed approach in this paper. The code is publicly available at https://github.com/Tony-Hao/GenX.

## Conclusions

This paper focused on gender, fundamental information in clinical trial for electrical prescreening to recruit appropriate participants. To facilitate transgender population recruitment, a virtual gender model was developed. An automated approach was further proposed for gender information extraction and gender summarization from unstructured clinical trial text. Based on 100,134 real clinical trials, our approach was compared with 20 machine learning algorithms. The results presented that our approach achieved the best performance using both widely adopted metrics and macro-averaged metrics, demonstrating the effectiveness of the approach in gender information processing.

## Abbreviations

EMR: Electronic medical record
LMT: Logistic model tree
NIH: National institutes of health
NLP: Natural language processing
TG: Transgender

## Footnotes

1 https://www.clinicaltrials.gov/

2 https://www.clinicaltrials.gov/ct2/show/NCT01880489?term=NCT01880489 &rank = 1

3
https://www.clinicaltrials.gov/ct2/show/NCT03270969?term=NCT03270969&rank=1

4
https://www.clinicaltrials.gov/ct2/show/NCT02356302?term=NCT02356302&rank=1

5
https://www.clinicaltrials.gov/ct2/show/NCT02356302?term=NCT02356302&rank=1

6
https://www.clinicaltrials.gov/ct2/show/NCT02356302?term=NCT02356302&rank=1

7
https://www.clinicaltrials.gov/ct2/show/NCT03467347?term=NCT03467347&rank=1

8
https://www.clinicaltrials.gov/ct2/show/NCT03234400?term=NCT03234400&rank=1

9
https://www.clinicaltrials.gov/ct2/show/NCT02704208?term=NCT02704208&rank=1

10
https://www.clinicaltrials.gov/ct2/show/NCT00231465?term=NCT00231465&rank=1

11
https://www.clinicaltrials.gov/ct2/show/NCT01023620?term=NCT01023620&rank=1

12
https://www.clinicaltrials.gov/ct2/show/NCT02401867?term=NCT02401867&rank=1

13
https://www.clinicaltrials.gov/ct2/show/NCT02410434?term=NCT02410434&rank=1

14
https://www.clinicaltrials.gov/ct2/show/NCT02518009?term=NCT02518009&rank=1

15
https://www.clinicaltrials.gov/ct2/show/NCT01869595?term=NCT01869595&rank=1

16
https://www.clinicaltrials.gov/ct2/show/NCT02573948?term=NCT02573948&rank=1
